# Supplementary material for: Saccharomyces boulardii (CNCM I-745) improves intestinal damage in sepsis by remodeling intestinal flora structure
Source: Front Cell Infect Microbiol. 2025 Jul 31;15:1602792. doi: 10.3389/fcimb.2025.1602792 (PMC12350329; doi:10.3389/fcimb.2025.1602792)
Supplement: Supplementary file 1 [file DataSheet1.zip › summary/summary/7_Differential_analysis/2_LEfSe_result/Group_AW4_vs_AW0_0.05_3.0/LEfSe_Group_AW4_vs_AW0.cladogram.pdf]

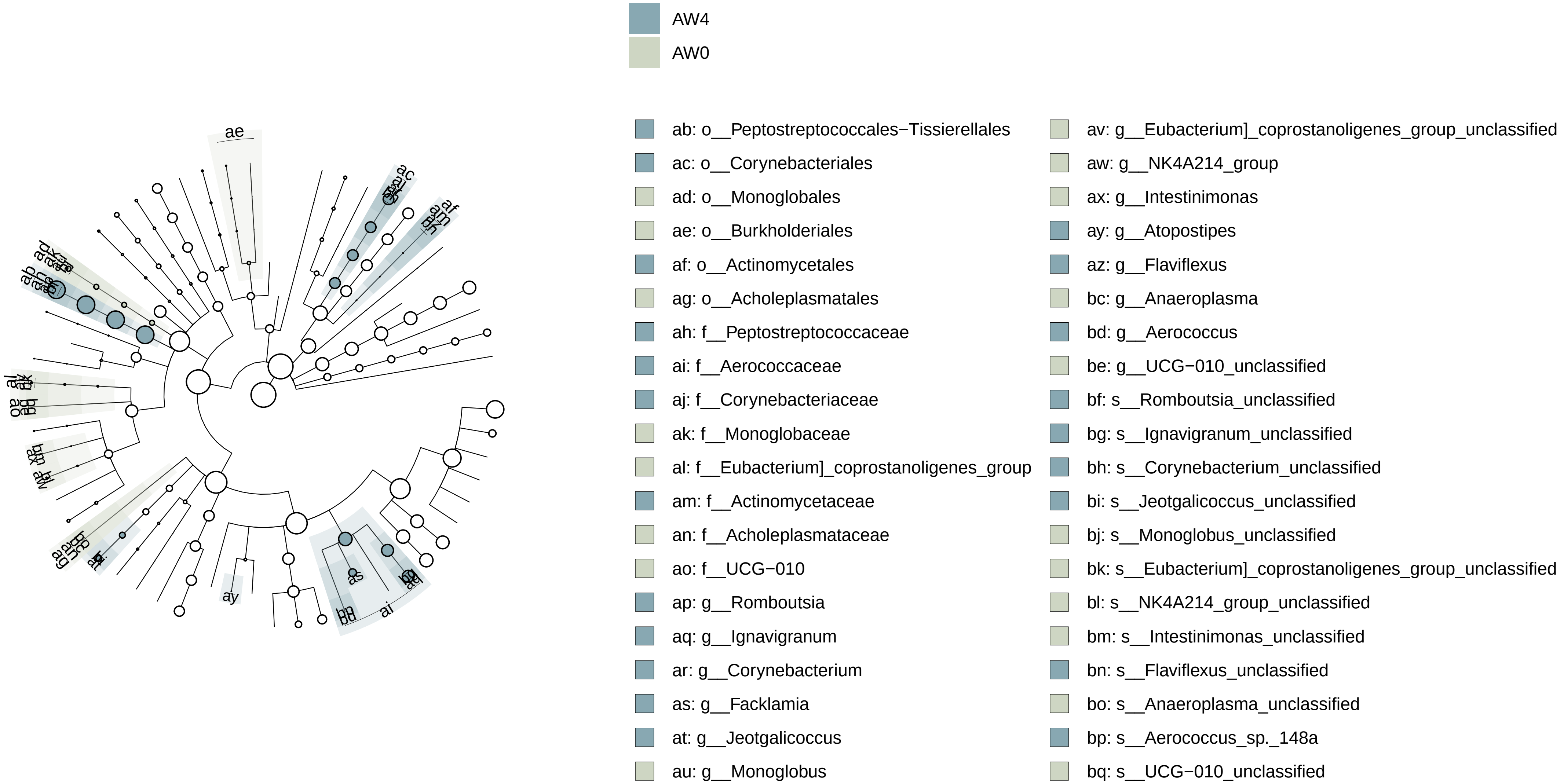
AW4
AW0
ab: o__Peptostreptococcales−Tissierellales
ac: o__Corynebacteriales
ad: o__Monoglobales
ae: o__Burkholderiales
af: o__Actinomycetales
ag: o__Acholeplasmatales
ah: f__Peptostreptococcaceae
ai: f__Aerococcaceae
aj: f__Corynebacteriaceae
ak: f__Monoglobaceae
al: f__Eubacterium]_coprostanoligenes_group
am: f__Actinomycetaceae
an: f__Acholeplasmataceae
ao: f__UCG−010
ap: g__Romboutsia
aq: g__Ignavigranum
ar: g__Corynebacterium
as: g__Facklamia
at: g__Jeotgalicoccus
au: g__Monoglobus
av: g__Eubacterium]_coprostanoligenes_group_unclassified
aw: g__NK4A214_group
ax: g__Intestinimonas
ay: g__Atopostipes
az: g__Flaviflexus
bc: g__Anaeroplasma
bd: g__Aerococcus
be: g__UCG−010_unclassified
bf: s__Romboutsia_unclassified
bg: s__Ignavigranum_unclassified
bh: s__Corynebacterium_unclassified
bi: s__Jeotgalicoccus_unclassified
bj: s__Monoglobus_unclassified
bk: s__Eubacterium]_coprostanoligenes_group_unclassified
bl: s__NK4A214_group_unclassified
bm: s__Intestinimonas_unclassified
bn: s__Flaviflexus_unclassified
bo: s__Anaeroplasma_unclassified
bp: s__Aerococcus_sp._148a
bq: s__UCG−010_unclassified
